# Drinking at Meals

**Published:** 1880-07

**Authors:** 


*Prenez garde*, "take care," "look out," "wide awake," you scissoring, careless, lazy, sleepy editors! Your negligence sometimes pulls down in a night what you have been for a long time building up. A taking title or an authoritative signature often gains admittance for an article which is not merely scientific nonsense, but downright and pernicious untruth. Only think of a dozen or so of our religious newspapers recommending beer and wine at meals as a positive advantage!

We have seen an article headed *Drinking at Dinner*, and credited to "*Orr's Chemistry*," going the rounds of our exchanges, ☞ *when we told them years ago* (*!*) that drinking at meals was a pernicious practice—even a draught of cold water, unless in high health, and even then, all that could be said of it is, that it may be done with impunity by the robust and the strong. A sip or two now and then during a meal may be allowable, especially if it be of some mild hot tea; but cold water, to that extent, even, is positively hurtful to all persons in feeble health, of small vitalities, of little bodily vigor. But here is the article verbatim:

"Not seldom do we hear the opinion advanced, that drinking during a meal is an obnoxious habit; but quite wrongfully; for the gastric juice may be diluted with a considerable quantity of water without losing its dissolving power in the slightest degree. Only a superabundance of water (!—*Ed.*) would diminish or arrest the peculiar action of the matters contained in the digestive fluids. Large draughts of water, therefore, will be the most injurious with aliments difficult of digestion, like the fats; and hence the drinking of too much water after fat pork, for instance, is properly avoided; but in countries where soup does not constitute a regular part of the meal, drinking water is positively to be recommended. Beer and wine at dinner are also hurtful only if taken in excess; for in the latter case the alcohol coagulates the albuminous substances, not only of the food, but also of the digestive fluids, and thus disturbs digestion. If taken in a moderate quantity, these beverages are calculated to cause the meal to hold out longer; for the fact that we are not so soon hungry again after a meal with wine than if we have taken only water with it, is to be accounted for by the slower combustion of the constituents of our body, inasmuch as the alcohol we have imbibed takes possession of the inhaled oxygen. Hence, wine with a meal is extremely useful when a long journey, or work in hand, renders it impossible to take food again at the usual time; so much the more so, as such detention from food itself usually causes an acceleration of the metamorphosis of the tissues, which beer and wine efficiently obviate."

To the above we reply, that actual looking into the stomach during the process of digestion authorizes the following observations:

1. The introduction of any cold liquid into the stomach *instantly* arrests the progress of digestion, and the suspension continues, until the liquid introduced has been there long enough to be warmed up to the natural temperature of the stomach, which is, one hundred degrees of Fahrenheit.

2. When *any* liquid is introduced into the stomach during digestion, the progress of digestion is arrested, and remains so, until the watery particles have been removed.

Now what can be the use of a man's talking against observed facts, meeting them with bare assertion?

He goes further and says that "alcoholic drinks are hurtful only if taken in excess." This sentiment is a dangerous one, even if it were true, for its latitude is boundless. *Only in excess!* What is the rule? Who is the judge?

"Wine or beer at meals are extremely useful because a person does not get hungry so soon!" Then we advise him to eat more, and let the wine and beer alone.

Another reason is, if you happen to be compelled to go longer without the next meal, "such detention from food causes an acceleration of the metamorphosis of the tissues." That sounds "*scientific*" certainly, whatever may be its sense or truth. But if you happened not to get another "horn" as soon as you expected, what is to prevent "an acceleration of the metamorphosis of the tissues"? What nonsense!

The practical meaning of the expression is, a man who drinks a glass or two of wine at dinner can work longer without another meal than his companion, who in all respects else was equal to him.

We beg leave to say that there is not a syllable of truth in any such statement. Any man of observation knows better. And yet this learned twaddle has been going the rounds of the religious press for months, one paper copying it from another Do our religious editors do their whole duty when they admit such articles without remark, without rebuke? The article attracts attention by recommending water, and ends by recommending wine. IN THE NAME OF THE PROPHET—*Figs!* exclaims the itinerant street vendor of Constantinople. Really, it seems to us very much like the work of a liquor dealer, who having made some jolly editor happy with a bottle of his best, secured a promise of insertion, that, stripped of all verbiage, means "a glass of wine at dinner is good for everybody," and he has found not a few temperance and religious editors to join in the acclaim, "*just so.*"

Wake up, ye gentlemen of the press! Your work is not yet done. Our poor-houses, and prisons, and penitentiaries, are too full of the friends of free drink for you to go to sleep so early in the day.

But let us tell you a fact before we close, which, perhaps, you may have forgotten, and may be, when that next bottle of the best comes round with another oiled untruth, you may be more on your guard. It's a little fact, just. It is an observed fact that a drink of brandy taken into the stomach before eating, *paralyzes it for several hours*, and that it does not regain its natural healthful condition for *thirty-six hours* afterwards; consequently digestion is impeded; and keep it impeded on a hearty meal for a time, and it will kill any man. If alcohol in any of its forms is taken only during meals or soon after, the ill results are modified.

